# Spontaneous Splenic Rupture following Colorectal Surgery and Hemodialysis

**DOI:** 10.1155/2019/8278419

**Published:** 2019-06-20

**Authors:** Ahmed Mohammed AlMuhsin, Antonio Privitera, Ameera Balhareth, Khalid Sabr

**Affiliations:** ^1^Department of General Surgery, King Fahd Military Medical Complex, Dammam, Saudi Arabia; ^2^Department of General Surgery, Colorectal Surgery, King Fahd Specialist Hospital Dammam, Dammam, Saudi Arabia

## Abstract

Atraumatic splenic rupture is rarely encountered in clinical practice compared to traumatic rupture. General risk factors include hematological, infectious, or malignant splenic diseases, uremic coagulopathy, use of heparin, hypertension, and immune-compromised status. Spontaneous splenic rupture following colorectal surgery has never been reported. Maintaining a high index of suspicion in patients presenting with left upper quadrant pain and tenderness is crucial. Diagnosis can be made with the aid of an ultrasound or CT scan. The management plan should be tailored to the patient's clinical conditions. The authors present a case of spontaneous splenic rupture in a patient following colectomy for cancer and undergoing postoperative hemodialysis and discuss the possible etiological factors.

## 1. Introduction

The spleen is the second most commonly injured organ following abdominal trauma [1]. Unlike traumatic splenic rupture, spontaneous splenic rupture is a rare entity that may be associated with various conditions including malignancies, splenic infarct, coagulopathies, anticoagulant therapy, portal hypertension, intrasplenic venous thrombosis, malaria, and focal splenic lesions [2].

Spontaneous splenic rupture in patients undergoing colorectal surgery has never been described. In the reported case, postoperative hemodialysis is deemed to have been the precipitating cause with only few cases reported in the literature. Although the exact etiology is still unclear, the use of heparin during hemodialysis, uremic coagulopathy, amyloidosis, infections, and splenic infarction could represent significant risk factors [3]. A high index of suspicion is paramount in patients undergoing hemodialysis and developing acute abdominal pain and hypovolemic shock in order to reduce morbidity and mortality.

## 2. Case Report

**Figure 1:**
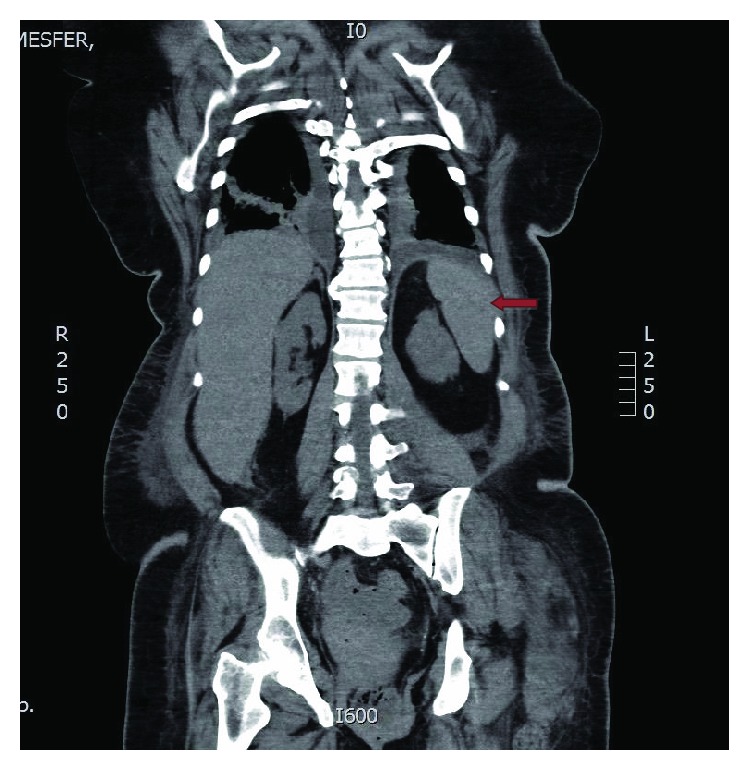
CT scan of the abdomen with IV contrast showing a normal spleen (red arrow).

A 61-year-old woman was referred by another institution with a diagnosis of distal transverse colon invasive adenocarcinoma and resectable liver metastases. Her past medical history revealed type 2 diabetes mellitus, hypertension, and chronic kidney disease. No relevant family history was noted. The case was discussed at the multidisciplinary tumor board meeting, and the decision was taken for the patient to undergo surgery for the primary tumor and subsequently address the treatment of liver metastases. A laparoscopic-assisted transverse colectomy with primary anastomosis was performed with no intraoperative complications. On the fourth postoperative day, she developed tachypnea, fever, and leukocytosis. A CT scan of the chest and abdomen with contrast was carried out. This showed a right middle lobe opacification consistent with pneumonia. A small <5 cm localized pelvic collection was noted; otherwise, the intra-abdominal organs including the spleen were unremarkable (Figure 1). Blood cultures were negative. Conservative treatment with intravenous ciprofloxacin was initiated. Her condition slowly improved. However, three days later, she developed severe metabolic acidosis due to acute renal failure. She was shifted to the ICU and underwent urgent hemodialysis. Four days after starting hemodialysis, she complained of acute left upper abdominal pain and developed hypovolemic shock.

Laboratory investigations showed normochromic normocytic anemia (hemoglobin 8.7 g/dL (12-17), white cell count 20.8 × 10^9^/L (4-11), platelet count 517 × 10^9^/L (150-400), blood urea nitrogen 12.9 mmol/L (2.7-7.2), and creatinine 342 *μ*mol/L (53-97)). Liver function tests showed an albumin of 14 g/L (34-50), alkaline phosphatase of 225 *μ*/L (54-144), prothrombin time of 16.1 (9.6-12.6), partial thromboplastin time of 36 sec. (24.3-30.2), and international normalized ratio of 1.5 (0.8-1.2).

Blood gases revealed a pH of 7.15 (7.3-7.4), pCO_2_ of 27 mmHg (36-46), HCO_3_ of 18.5 mmol/L (21-28), lactate of 1.5 mmol/L (0.5-1), and anion gap of 24.2.

**Figure 2:**
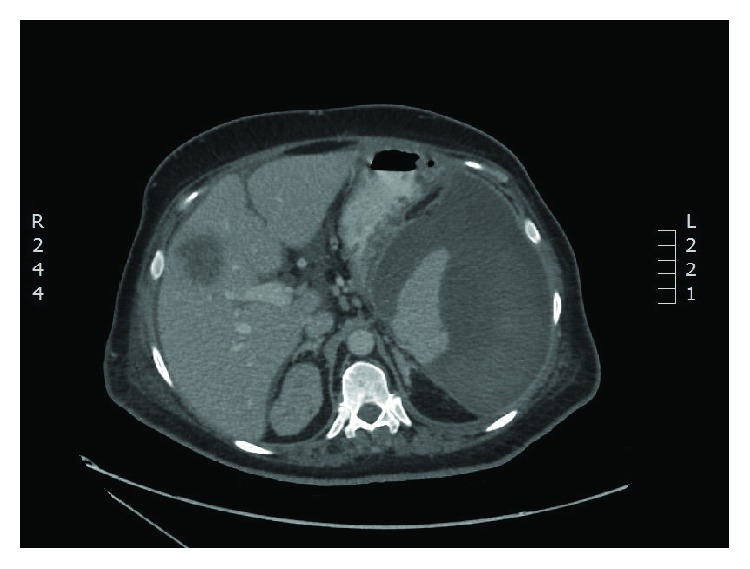
CT scan of the abdomen with IV contrast showing a large subcapsular splenic hematoma with gas formation.

A CT scan of the abdomen and pelvis showed a large subcapsular splenic hematoma with gas formation. There was no evidence of bowel perforation or obstruction (Figure 2).

An emergency laparotomy was performed, and this showed a ruptured splenic hematoma. Peritoneal lavage and splenectomy were performed. Histology showed a necrotic spleen with fibrin and focal fat necrosis with no evidence of malignancy.

The patient made a slow but uneventful recovery and was eventually transferred to the nephrology unit to continue treatment for renal failure.

## 3. Discussion

The most common cause of splenic rupture is abdominal trauma [1]. Spontaneous rupture of the spleen in the absence of trauma or underlying pathology is rare to a degree that some authors debate its existence. However, many cases have been reported suggesting that spontaneous rupture can occur in a normal spleen [2]. Conversely, pathological splenic rupture occurs due to underlying diseases including hematological, infectious, or malignant infiltration [3, 4].

Early diagnosis and management is crucial as it is a life-threatening condition with a mortality up to 12% [4]. Diagnosis is aided by US or CT scan imaging. Signs of splenic rupture on the CT scan include nonenhancing foci of hyperdensity or hypodensity and intracapsular or intraperitoneal fluid [5, 6].

Few cases have been reported in the literature of spontaneous splenic rupture in hemodialysis patients [3, 7, 8]. There are many risk factors including the use of heparin, amyloidosis, the immune-compromised status of patients that makes them susceptible to infections, and uremic coagulopathy [3, 7]. The latter is thought to be the main cause of spontaneous splenic rupture in hemodialyzed patients [3, 9].

Patients on dialysis are likely to have an abnormal coagulation profile. This may be evident in the form of prolonged bleeding time, platelet dysfunction, impaired platelet aggregation, decreased activity of platelet factor III, and impaired prothrombin consumption [7, 9]. Patients may show a fibrinolysis defect at the level of the plasminogen that may contribute to the development of atherosclerosis and thrombosis and subsequent complications. Hypertension and volume overload are other risk factors [7].

Splenic artery calcification that can be secondary to uremia and can alter arterial stability has been considered a risk factor for splenic rupture [10].

As most of the cases reported, our patient received anticoagulation during hemodialysis and had evident uremic coagulopathy. There were no other risk factors such as trauma, malignancy, or amyloid deposition. It is possible that pneumonia might have been a contributing factor; however, blood cultures were unremarkable. A first postoperative CT scan had shown a normal spleen ruling out possibility of inadvertent injury during the laparoscopic procedure for cancer. A conservative approach in the patient was deemed inappropriate in view of her instability and evidence of infection with a gas forming organism on preoperative scan.

## 4. Conclusion

Spontaneous splenic rupture is a rare entity that can be associated with many clinical conditions including malignant, hematological, and infectious causes. It is a fatal condition that can be missed easily especially in the absence of trauma. Prompt diagnosis and management is essential for better patient outcome. Clinicians should have a high index of suspicion in patients with uremia and hemodialysis when presenting with left upper abdominal pain and tenderness, even in the postoperative period. The decision of conservative or operative management should depend on the benefit-risk analysis for each single case.
